# Assessing ADME gene coverage: An observational study on chloroquine therapy for COVID-19

**DOI:** 10.1097/MD.0000000000044340

**Published:** 2025-09-12

**Authors:** Nabil Zaid, Lamyaa Benchikhi, Naoual El Abboudi, Loubna Khalki, Oussama Badad, Youness Limami, Abdallah Naya, Mounia Oudghiri, Banacer Himmi, Younes Zaid

**Affiliations:** a Higher Institute of Nursing and Health Technical Professions (ISPITS), Rabat – Ministry of Health and Social Protection, Rabat, Morocco; b Biology Department, Faculty of Sciences, Mohammed V University in Rabat, Rabat, Morocco; c Research Center, Mohammed VI University of Health Sciences (UM6SS), Casablanca, Morocco; d Southern Illinois University, Carbondale, IL; e Research Center of Abulcasis University of Health Sciences, Rabat, Morocco; f Immunology and Biodiversity Laboratory, Department of Biology, Faculty of Sciences Ain Chock, Hassan II University, Casablanca, Morocco.

**Keywords:** ADME genes, chloroquine, COVID-19, genotyping platforms, personalized medicine, pharmacogenomics

## Abstract

This observational study aimed to compare various genotyping and enrichment platforms to determine the most comprehensive coverage for genome-wide association studies, specifically targeting new therapeutic approaches against coronaviruses using chloroquine. Pharmacogenomic studies have become essential for understanding individual drug responses, and optimal platform selection is critical for identifying relevant genetic variants. We developed Python scripts to assess the coverage rates of these platforms, focusing on the absorption, distribution, metabolism, and excretion (ADME) genes involved in drug absorption, distribution, metabolism, and excretion. Additionally, the PLINK tool was employed to evaluate single nucleotide polymorphisms in linkage disequilibrium with ADME variants, providing insights into the extended coverage achieved through correlation with these variants. Among the genotyping platforms analyzed, Axiom genotyping and SureSelect enrichment platforms demonstrated the most extensive coverage of the genome and key pharmacogenomic regions. These platforms effectively captured a significant proportion of ADME gene variants, which are crucial for predicting individual responses to chloroquine. The extensive coverage provided by the Axiom and SureSelect platforms supports their use in the design of pharmacogenomic studies, potentially revealing new therapeutic targets for combating coronaviruses, including through the use of chloroquine treatment. The results highlight the importance of selecting appropriate genotyping and enrichment technologies for maximizing the impact of pharmacogenomic research.

## 1. Introduction

Recent studies have underscored the effectiveness of chloroquine against coronavirus.^[1,2]^ However, the imperative consideration of the reliability and efficacy of statistical data before administering treatments to patients often creates a void despite these promising findings.

At present, there is no definitively proven pharmacological treatment for severe acute respiratory syndrome coronavirus 2. Recent publications have shed light on the potential benefits of chloroquine, a drug widely used worldwide, in managing patients infected with the novel coronavirus.^[1,2]^ Chloroquine has been extensively used in the treatment of malaria, amebiosis, human immunodeficiency virus, and autoimmune conditions.^[3]^

Chloroquine blocks viral infection by increasing endosomal pH and interfering with terminal glycosylation of the ACE2 receptor, which can affect severe acute respiratory syndrome coronavirus 2 binding.^[3,4]^ In clinical trials conducted in China and a nonrandomized trial of coronavirus disease 2019 (COVID-19) patients in France, chloroquine has shown greater clinical efficacy than controls.^[1,5]^ Additional information on the safety and effectiveness of chloroquine is critical for clinicians’ decisions to save lives during the global battle against COVID-19.

The variability of responses to drug treatments and the prediction of their adverse effects depend on a set of environmental, cultural, and genetic factors. Several studies have highlighted the genetic factors that are responsible for the great interindividual variability in the pharmacokinetics and pharmacodynamics of drugs. Pharmacogenomic studies have contributed to clarifying interindividual variations in drug responses. An in-depth understanding of these findings opened up new therapeutic pathways and disclosed new prospects toward personalized approaches.

Genetic variations correlated with drug absorption, distribution, metabolism, and excretion (ADME) contribute to the prediction of drug response and prevention of adverse drug reactions. Drug safety and effectiveness, determined by the concentration and effective site, remain dependent on ADME mechanisms.^[6–8]^ Since the CYP2C8, CYP2D6, and CYP3A ADME genes are involved in the drug response to chloroquine, we were interested in studying the variations in these genes.

Pharmacogenetic approaches to discover therapeutic targets by studying human genetic polymorphisms and their association with diseases have achieved impressive progress toward the personalization of pharmaceutical treatment, with more than 100 drugs on the US list, generally in collaboration with academic researchers and clinicians. The Food and Drug Administration has established recommendations for genetic testing to ensure drug safety and efficacy.^[9]^

The complexity of gene interactions and therapeutic pathways while using current commercial platforms does not allow appropriate handling and coverage of ADME variants.^[10]^ In this context, the current study aimed to calculate and analyze the coverage rates of the CYP2C8, CYP2D6, and CYP3A ADME genes for each of the variants and amplicon lists from our collection. Hence, we used the chromosome and position of each variant from the ADME lists, according to the Hg19 construct.

We calculated the coverage of our interest lists using scripts written in Python according to the following formula:


$$\begin{array}{l} \%Coverage=\left(Interestlist\cap ADMElist\right)\times 100/ADMElist. \end{array}$$

We also considered coverage of the markers of interest that can be achieved by markers in linkage disequilibrium (LD). To refine our evaluation and improve relevance, we compared the expected theoretical results according to the targeted coverage of the different technologies with the practical results obtained. The results of this analysis allowed us to determine which interest lists covered the best ADME genes.

## 2. Materials and methods

### 2.1. Study design

This observational study aims to evaluate the genetic coverage of genotyping and enrichment platforms for ADME variants in the context of chloroquine treatment. No interventions were performed on subjects, and the analysis focused on evaluating platform performance in identifying relevant variants from existing genotyping data.

### 2.2. Participants and data sources

The data used in this study were obtained from the 1000 Genomes Project Phase 3, accessible via the International Genome Sample Resource (ftp://ftp.1000genomes.ebi.ac.uk). We selected publicly available VCF files representing 3 population groups (European ancestry population, African ancestry population, and East Asian ancestry population), which were then converted into PLINK format for downstream analysis. These datasets provided comprehensive genotyping information to evaluate ADME variant coverage. No new data were collected, and no direct human participants were involved in this study.

### 2.3. Bias management

To minimize selection bias, datasets were chosen to include a wide range of genetic diversity, representing different populations. The inclusion of multiple genotyping platforms helps to reduce biases related to a single analytical method. Furthermore, the selection criteria for ADME variants were consistent across all platforms studied.

### 2.4. Statistical analysis

Statistical analyses were conducted using Python scripts and PLINK, which allowed the assessment of single nucleotide polymorphism (SNP) coverage in LD with ADME variants. No missing data were observed in the datasets analyzed. Results were validated using standard statistical tests to ensure robustness and reproducibility.

### 2.5. Data

We have 2 sets of lists, ADME lists and interest lists (Table 1), which we used to calculate the coverage rates.

**Table 1:** ADME and interest lists used in this study.

ADME lists	Interest lists	
	Genotyping	Enrichment
ADME core (34 variants)	Omni (1S, 2.5S, 2.5, and 5S)	SureSelect V5 (~21,000 genes)
	Axiom (~85,500 variants)	HaloPlex (~21,000 genes)

ADME = absorption, distribution, metabolism, and excretion.

ADME variants are extracted from genes that have been determined to be associated with drug metabolism.^[11]^ The 34 variants used in this study were extracted from the CYP2C8, CYP2D6, and CYP3A genes, and the main isoforms affected or involved in the metabolism of chloroquine.^[12]^ In this observational study, we focused on the genotyping lists (Omni and Axiom) and enrichment lists (SureSelect and HaloPlex). These platforms are likely to cover the ADME genes.

### 2.6. Genotyping platforms

#### 2.6.1. Omni coverage

The Omni lists contain variants obtained via different Illumina genotyping chips.^[13]^ We have 4 Omni lists (1S, 2.5S, 2.5, and 5.0) obtained from Illumina’s FTP website: http://www.illumina.com/forms/ftp.ilmn. These lists contain 1 million variants for Omni 1S, 2 million variants for Omni 2.5S and Omni 2.5, and 5 million variants for Omni 5.0.

#### 2.6.2. Axiom coverage

The Axiom platform is designed by Affymetrix for genotyping large sample collections, such as those screened in biobanks, genome centers, and core labs. These arrays incorporate multiple content categories, including a Genome-Wide Association Study Panel of markers for genome coverage in major ethnic groups, rare coding SNPs and indels for exome analysis, pharmacogenomic markers, eQTLs, and newly discovered loss-of-function variants, including sequence insertions and deletions from recent exome sequencing initiatives.^[14]^ Axiom was included in our comparison due to its integrated pharmacogenomic content.

### 2.7. Enrichment platforms

#### 2.7.1. SureSelect coverage

The list SureSelect covers polymorphisms from the “Agilent Technologies” product to capture hybridization, which has the following website: http://www.genomics.agilent.com/. This list included 554,751 amplicons that were likely to cover the variants of each ADME list.

#### 2.7.2. HaloPlex coverage

The HaloPlex technology delivers outstanding performance, streamlined workflow, and low sample input requirements for next-generation human exome sequencing. The HaloPlex Exome was optimized to provide comprehensive coverage of the coding regions of the human genome.^[15]^

### 2.8. Linkage disequilibrium

LD, which refers to the nonassociation of alleles at different loci, serves as a key method for evaluating the connection between genomic variability and phenotypic traits in unrelated individuals. When a correlation is detected between a genotyped marker and a phenotype, it may either reflect a direct causal relationship or the marker LD with a nearby causal variant.^[16]^

To evaluate whether ADME variants not directly present on genotyping platforms could still be captured through correlated markers, we conducted LD-based coverage analysis using the PLINK software. LD was calculated using an *r*^2^ threshold of ≥0.8, a commonly accepted cutoff in pharmacogenomic studies for strong linkage, as it reflects a high degree of correlation sufficient for tagging untyped functional variants.^[8]^ This threshold balances sensitivity and specificity in identifying meaningful proxy variants.

The LD analysis was performed using the following PLINK command:


$$\begin{array}{l} plink--bf\;ilemydata--show\text{-}tagsmysnps.txt \end{array}$$

where “mysnps.txt” is a list of the 34 core ADME variant rsIDs. LD calculations were conducted using phased genotype data from the 1000 Genomes Project Phase 3 (European ancestry population, African ancestry population, East Asian ancestry population populations).

This approach allowed us to assess the extent to which each genotyping platform could indirectly capture ADME variants through nearby SNPs in high LD.

## 3. Results

To calculate the coverage, we relied on the variants contained in each genotyping chip. The physical coverage rates of the CYP2C8, CYP2D6, and CYP3A ADME genes by different genotyping platforms are summarized in Table 2. Among these platforms, Axiom and Omni 5.0 demonstrated the highest coverage of the core variant list.

**Table 2:** The physical coverage of CYP2C8, CYP2D6, and CYP3A pharmacogenetic variants by genotyping chips.

	Omni 1S	Omni 2,5S	Omni 2,5	Omni 5	Axiom
Physical coverage	1/34 2.94%	4/34 11.76%	5/34 14.71%	8/34 23.53%	18/34 52.94%

To assess whether coverage could be further improved, we evaluated the contribution of LD in capturing additional variants from the ADME lists (Table 3). While the inclusion of SNPs in LD with ADME variants slightly increased coverage for Omni platforms, the overall improvement remained suboptimal. However, for the Axiom platform, considering LD significantly enhanced the coverage rate, reaching 88.24% (30 out of 34 variants).

**Table 3:** The coverage of CYP2C8, CYP2D6, and CYP3A pharmacogenetic variants by genotyping chips when considering LD.

	Omni 1S	Omni 2.5S	Omni 2.5	Omni 5	Axiom
Coverage with LD	2/34 5.88%	14/34 41.18%	10/34 29.41%	20/34 58.82%	30/34 88.24%

LD = linkage disequilibrium.

**Figure 1.**
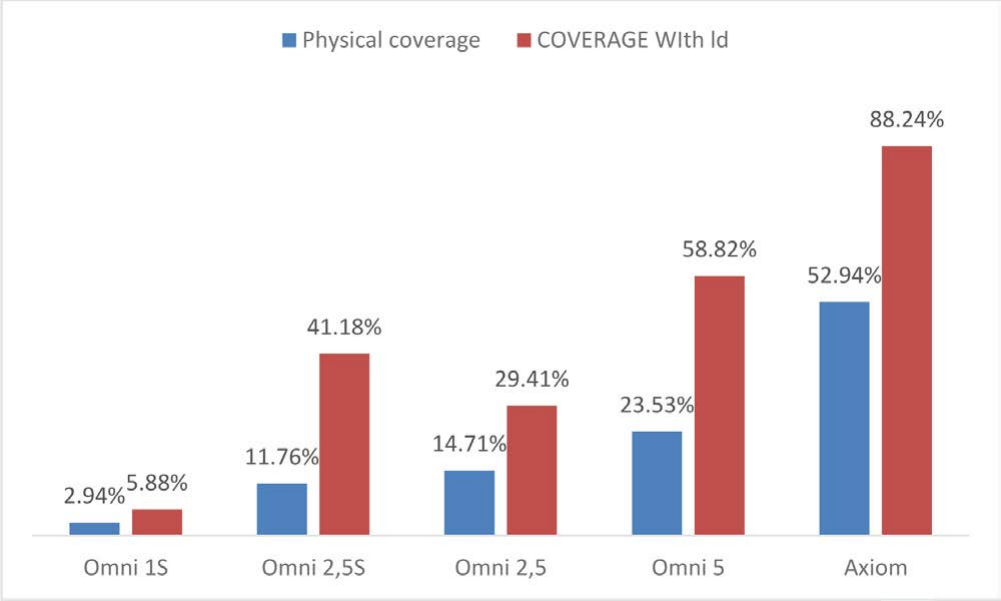
Comparison of physical coverage rates and linkage disequilibrium (LD)-adjusted coverage rates across Omni (1S, 2.5S, 2.5, 5) and Axiom genotyping platforms. Physical coverage represents the direct proportion of pharmacogenomic variants within CYP2C8, CYP2D6, and CYP3A genes captured by each genotyping array. LD-adjusted coverage accounts for additional variants indirectly captured due to their high correlation (LD threshold: *r*^2^ ≥ 0.8) with directly genotyped variants. Higher coverage rates indicate greater effectiveness of the platform for capturing relevant pharmacogenomic variants.

These findings are illustrated in Figure 1, which compares the physical coverage of genotyping platforms against their LD-adjusted coverage. The results highlight that while LD improves coverage for certain platforms, it remains insufficient for achieving full variant capture.

**Figure 2.**
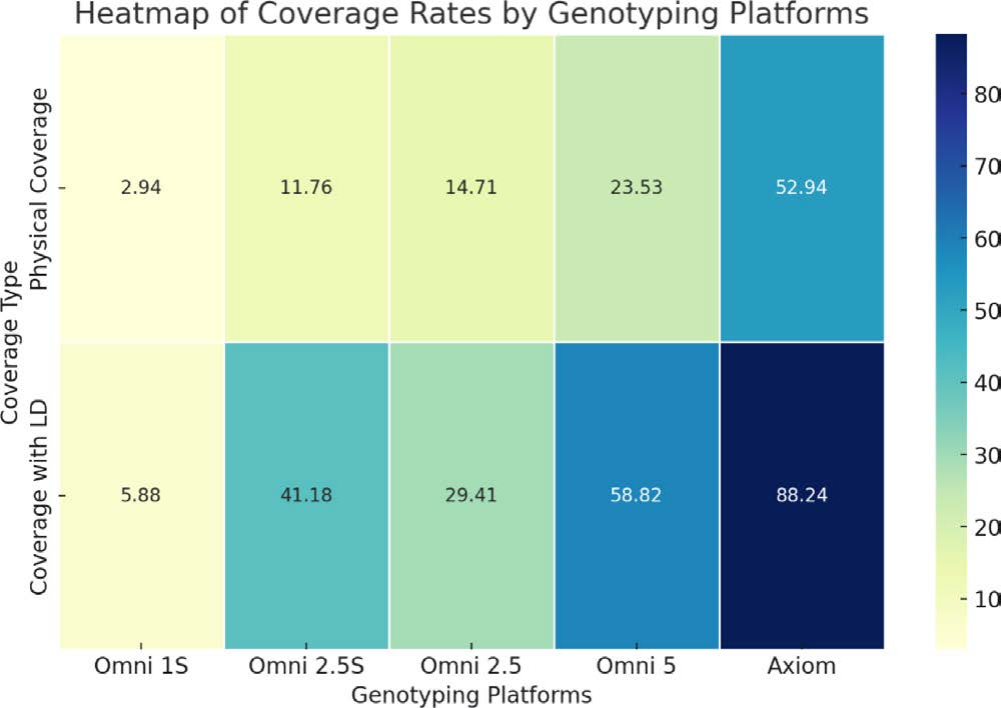
Heatmap illustrating coverage rates of ADME variants (CYP2C8, CYP2D6, and CYP3A) across the studied genotyping platforms (Omni 1S, Omni 2.5S, Omni 2.5, Omni 5, and Axiom). Colors represent coverage rates, ranging from low (cool colors) to high (warm colors), providing an intuitive visual comparison of the relative effectiveness of each platform in capturing critical genetic variants involved in pharmacological response to chloroquine.

As shown in Figure 2, a heatmap of coverage rates across different genotyping technologies visually represents these variations, emphasizing the superior performance of Axiom when LD is accounted for.

Despite these improvements, even when combining all 5 platforms and considering LD, 2 variants (rs72549353 and rs72549357) remain uncovered (Table 4), underscoring the limitations of current genotyping technologies.

**Table 4:** Summary table of genotyping platforms coverage.

Gene	rsID	Chr:Pos	Omni 1S	Omni 2.5S	Omni 2.5	Omni 5	Axiom	Omni 1S (LD)	Omni 2.5S (LD)	Omni 2.5 (LD)	Omni 5 (LD)	Axiom (LD)
CYP3A5	rs776746	7:99270539	0	0	1	1	1	0	0	1	1	1
CYP3A5	rs10264272	7:99262835	0	0	1	1	1	0	0	1	1	1
CYP3A5	rs41279854	7:99247772	0	0	0	0	1	0	0	0	0	1
CYP3A5	rs41303343	7:99250393	0	0	0	0	1	0	0	0	0	1
CYP3A5	rs55965422	7:99264573	0	1	0	1	1	0	1	0	1	1
CYP3A4	rs2242480	7:99361466	1	1	1	1	1	1	1	1	1	1
CYP3A4	rs4646438	7:99364034	0	0	0	0	0	0	1	0	1	0
CYP3A4	rs55785340	7:99365983	0	0	0	0	1	0	0	0	0	1
CYP3A4	rs67666821	7:99355806	0	0	0	0	0	0	1	0	1	1
CYP2D6	rs1065852	22:42526694	0	0	0	0	1	0	0	0	0	1
CYP2D6	rs1080985	22:42528382	0	0	0	0	1	0	0	0	0	1
CYP2D6	rs3892097	22:42524947	0	0	0	0	1	0	0	0	0	1
CYP2D6	rs5030655	22:42525086	0	0	0	0	1	0	0	0	0	1
CYP2D6	rs5030656	22:42524178	0	0	0	0	0	0	0	1	0	1
CYP2D6	rs5030862	22:42526670	0	1	0	1	0	0	1	0	1	0
CYP2D6	rs5030863	22:42539583	0	0	0	0	0	0	1	0	1	1
CYP2D6	rs5030865	22:42525035	0	0	0	0	0	1	1	1	1	1
CYP2D6	rs5030867	22:42523858	0	0	0	0	1	0	0	0	0	1
CYP2D6	rs28371706	22:42525772	0	0	0	0	1	0	0	0	0	1
CYP2D6	rs28371725	22:42523805	0	0	0	1	1	0	0	0	1	1
CYP2D6	rs35742686	22:42524244	0	0	0	0	1	0	0	0	0	1
CYP2D6	rs72549346	22:42523534	0	0	0	0	0	0	1	0	1	1
CYP2D6	rs72549347	22:42527305	0	0	0	0	0	0	1	0	1	1
CYP2D6	rs72549349	22:42523843	0	0	0	0	0	0	1	0	1	1
CYP2D6	rs72549351	22:42524206	0	0	0	0	0	0	1	0	1	1
CYP2D6	rs72549352	22:42524214	0	0	0	0	0	0	0	0	0	0
CYP2D6	rs72549353	22:42527956	0	0	0	0	0	0	1	0	1	1
CYP2D6	rs72549354	22:42524820	0	0	0	0	0	0	1	1	1	1
CYP2D6	rs72549357	22:42526657	0	0	0	0	0	0	0	0	0	0
CYP2C8	rs1058930	10:96818119	0	0	1	1	1	0	0	1	1	1
CYP2C8	rs10509681	10:96798749	0	1	0	0	1	0	1	0	0	1
CYP2C8	rs11572103	10:96818106	0	0	1	1	1	0	0	1	1	1
CYP2C8	rs72558195	10:96824643	0	0	0	0	0	0	0	1	1	1
CYP2C8	rs72558197	10:96826973	0	0	0	0	0	0	0	1	1	1
**Total**			**1**	**4**	**5**	**8**	**18**	**2**	**14**	**10**	**20**	**30**

Bold values indicate the cumulative number of variants covered by each genotyping platform.

For enrichment platforms, we relied on the probe content of each list. The ADME gene coverage rate for the HaloPlex platform is presented in Table 5, and the coverage for the SureSelect platform is presented in Table 6. Among the evaluated platforms, SureSelect demonstrated the best coverage, capturing 33 out of 34 variants (97.05%), making it a suitable tool for pharmacogenomic studies. However, due to technological constraints, certain complex genomic regions, including specific ADME genes, are often excluded from high-throughput genotyping and sequencing chips.^[17]^

**Table 5:** Coverage rate of CYP2C8, CYP2D6, and CYP3A pharmacogenetic variants by HaloPlex.

Target ID	Regions	Coverage	High coverage (≥90%)	Low coverage (<90%)
CYP2C8	5	100%	5	0
CYP2D6	20	85%	17	3
CYP3A4	4	75%	3	1
CYP3A5	5	100%	5	0

**Table 6:** Coverage rate of CYP2C8, CYP2D6, and CYP3A pharmacogenetic variants by SureSelect.

Target ID	Regions	Coverage	High coverage (≥90%)	Low coverage (<90%)
CYP2C8	5	100%	5	0
CYP2D6	20	95%	19	1
CYP3A4	4	100%	4	0
CYP3A5	5	100%	5	0

### 3.1. Missing data

No missing data were observed in the datasets analyzed during this study. All genotyped variants and relevant data points were included in the final analysis.

## 4. Discussion

Although clustering quality generally suffices for the majority of genetic variants found on commercial platforms, it is not uncommon for clustering to falter in highly homologous genomic regions, such as those encompassing several ADME genes, such as CYP2C8, CYP2D6, CYP3A4, and CYP3A5.^[8]^ Previous studies have highlighted the limitations of genome-wide approaches for pharmacogenomic testing. Weinshilboum et al^[18]^ focused on a set of genes critical to pharmacogenomics and personalized medicine, utilizing only genotyping platforms. Their findings indicated that even when considering SNPs in LD, genotyping platforms inadequately covered these genes. Another study^[7]^ evaluated sequencing platforms and revealed that the HaloPlex enrichment platform provided the most comprehensive coverage of ADME variants. However, this coverage still falls short for all ADME genes.

Additionally, there are commercially available chips containing targeted pharmacogenetic content, such as the DMET panel (enzymes and transporters metabolizing the metabolism of Affymetrix) or the iPLEX PGx Pro panel from Agena, which offers focused coverage of challenging pharmacogenetic variants. However, these panels were not included in this study as they lacked genomic coverage, yet they could potentially serve as supplementary tests to augment a genomic set in pharmacogenomic investigations.^[19]^ The Axiom genotyping and SureSelect enrichment platforms provide both genome-wide and pharmacogene coverage, which are essential for uncovering new variants associated with adverse drug reactions. This combined coverage, demonstrating the most comprehensive representation of the core list, will aid in shaping pharmacogenomic research designs and will likely facilitate the discovery of new therapeutic targets in the fight against coronavirus through chloroquine treatment.

Despite the strengths of this study, several limitations should be acknowledged. First, the study was limited by the use of preexisting datasets, which may not fully represent all populations, particularly those underrepresented in genetic studies. This could affect the generalizability of the findings to broader populations. Additionally, the sample size of the datasets, though diverse, may not capture the full spectrum of genetic variation relevant to ADME genes, especially rare variants. Another potential limitation is that the study did not include an analysis of real-world clinical outcomes, which could provide further insight into the pharmacogenomic relevance of the identified variants.

In terms of strengths, this study offers a robust evaluation of ADME gene coverage across multiple genotyping and enrichment platforms, using state-of-the-art tools such as Python and PLINK to assess SNPs in LD. The use of multiple platforms reduces bias associated with reliance on a single technology, and the consistency of the variant selection criteria ensures that the results are comparable across datasets. Furthermore, the study’s focus on pharmacogenomics in the context of chloroquine treatment for COVID-19 highlights the potential for future therapeutic applications.

## 5. Conclusion

Our findings underscore the importance of comprehensive coverage in pharmacogenomic studies, particularly in the context of COVID-19 treatment for chloroquine. Despite advancements in genotyping and enrichment platforms, challenges remain, particularly in highly homologous genomic regions. Although targeted panels show potential for focused coverage, they may not offer the breadth required for genome-wide studies. The Axiom and SureSelect platforms have emerged as promising options, providing extensive coverage that could significantly impact pharmacogenomic research and aid in the discovery of new therapeutic targets for combating coronavirus with chloroquine treatment.

## Author contributions

**Conceptualization:** Nabil Zaid, Oussama Badad, Younes Zaid.

**Data curation:** Nabil Zaid, Lamyaa Benchikhi, Naoual El Abboudi, Youness Limami.

**Formal analysis:** Nabil Zaid, Naoual El Abboudi, Loubna Khalki, Oussama Badad, Youness Limami.

**Investigation:** Younes Zaid.

**Methodology:** Nabil Zaid, Lamyaa Benchikhi, Younes Zaid.

**Project administration:** Nabil Zaid.

**Resources:** Nabil Zaid, Naoual El Abboudi, Loubna Khalki, Banacer Himmi, Younes Zaid.

**Software:** Nabil Zaid, Naoual El Abboudi.

**Supervision:** Nabil Zaid, Abdallah Naya, Mounia Oudghiri, Banacer Himmi, Younes Zaid.

**Validation:** Nabil Zaid, Naoual El Abboudi, Oussama Badad, Abdallah Naya, Mounia Oudghiri, Banacer Himmi, Younes Zaid.

**Visualization:** Nabil Zaid, Abdallah Naya, Mounia Oudghiri, Younes Zaid.

**Writing – original draft:** Nabil Zaid, Lamyaa Benchikhi, Loubna Khalki, Oussama Badad, Younes Zaid.

**Writing – review & editing:** Nabil Zaid, Lamyaa Benchikhi, Banacer Himmi, Younes Zaid.
